# Effects of Cell Grafting on Coronary Remodeling After Myocardial Infarction

**DOI:** 10.1161/JAHA.113.000202

**Published:** 2013-06-21

**Authors:** Jill J. Weyers, Stephen M. Schwartz, Elina Minami, Dara D. Carlson, Sarah K. Dupras, Kevin Weitz, Michael Simons, Timothy C. Cox, Charles E. Murry, William M. Mahoney

**Affiliations:** 1 Department of Pathology, Center for Cardiovascular Biology and Institute for Stem Cell and Regenerative Medicine, University of Washington, Seattle, WA (J.J.W., S.M.S., E.M., D.D.C., S.K.D., K.W., C.E.M., W.M.M.); 2 Department of Medicine/Cardiology, University of Washington, Seattle, WA (E.M., C.E.M.); 3 Department of Bioengineering, University of Washington, Seattle, WA (C.E.M.); 4 Division of Craniofacial Medicine, Department of Pediatrics, University of Washington, Seattle, WA (T.C.C.); 5 Center for Tissue and Cell Sciences, Seattle Children's Research Institute, Seattle, WA (T.C.C.); 6 Section of Cardiovascular Medicine, Department of Internal Medicine, Yale University School of Medicine, New Haven, CT (M.S.); 7 Department of Cell Biology, Yale University School of Medicine, New Haven, CT

**Keywords:** coronary angiography, grafting, myocardial infarction, myocardial revascularization, vascular remodeling

## Abstract

**Background:**

With recent advances in therapeutic applications of stem cells, cell engraftment has become a promising therapy for replacing injured myocardium after infarction. The survival and function of injected cells, however, will depend on the efficient vascularization of the new tissue. Here we describe the arteriogenic remodeling of the coronary vessels that supports vascularization of engrafted tissue postmyocardial infarction (post‐MI).

**Methods and Results:**

Following MI, murine hearts were injected with a skeletal myoblast cell line previously shown to develop into large grafts. Microcomputed tomography at 28 days postengraftment revealed the 3‐dimensional structure of the newly formed conducting vessels. The grafts elicited both an angiogenic response and arteriogenic remodeling of the coronary arteries to perfuse the graft. The coronaries upstream of the graft also remodeled, showing an increase in branching, and a decrease in vascular density. Histological analysis revealed the presence of capillaries as well as larger vascular lumens within the graft. Some graft vessels were encoated by smooth muscle α‐actin positive cells, implying that vascular remodeling occurs at both the conducting arterial and microvascular levels.

**Conclusions:**

Following MI and skeletal myoblast engraftment, the murine coronary vessels exhibit plasticity that enables both arteriogenic remodeling of the preexisting small branches of the coronary arteries and development of large and small smooth muscle encoated vessels within the graft. Understanding the molecular mechanisms underlying these 2 processes suggests mechanisms to enhance the therapeutic vascularization in patients with myocardial ischemia.

## Introduction

Repairing and replacing injured myocardium is a central goal in the treatment of myocardial infarction (MI). One promising method for achieving functional improvement following MI is to engraft cells into the injured area of the heart to replace the damaged myocardial tissue.^1–3^ Cardiomyocyte engraftment is difficult, in part because the vast majority of cardiomyocytes retained within the infarct fail to survive, resulting in small grafts.^4–5^ This poor survival is generally attributed to continued ischemia within the graft and surrounding tissue.^4,6^ Thus, if cell grafts are to restore mechanical function to the injured heart wall, they must become vascularized, thereby ensuring optimal survival.

Previous work involving cardiac grafts has revealed that many of the vessels within tissue grafts originate from the host.^3^ It is unclear, however, if the coronary network is sufficiently plastic to develop the hierarchical vasculature necessary to support a tissue graft postinfarction. Previous efforts to revascularize the injured myocardium have focused on angiogenic mechanisms.^7–10^ Angiogenesis, however, only provides new capillaries, and is therefore insufficient for establishing a hierarchically branched network capable of delivering blood efficiently.^11–14^ Thus, the vascularization of cell grafts requires a balance between angiogenesis and arteriogenic remodeling to organize vessels into a hierarchical branched network. This study is designed to characterize the coronary vascular growth and remodeling events that support the vascularization of myocardially implanted cell grafts post‐MI.

Our study utilized implanted skeletal muscle cells as a model. In contrast to cardiomyocyte engraftment, engrafted skeletal muscle myoblasts survive to form large metabolically active grafts within the infarcted heart.^15–16^ Although the skeletal muscle cells cannot electromechanically couple with the host myocardium^15,17–18^ and are therefore not a therapeutic option, the large size of these grafts implies that capillaries are formed within the graft and the new tissue is functionally perfused. Thus, skeletal myoblast grafts provide an excellent model in which to study the successful vascularization of cardiac grafts. Using a previously established model of MI and skeletal myoblast engraftment^15–16^ followed by microcomputed tomography (μCT) and histological analysis, we demonstrate that in order to perfuse the large tissue graft the coronary vasculature undergoes “arteriogenic remodeling”—the enlargement and remodeling of capillaries and preexisting nascent vessels into larger arterioles and arteries.

## Methods

### Cell Culture

C2C12 cells (ATCC, CRL‐1772) were cultured in high glucose Dulbecco's modified Eagle's medium (DMEM) media with 20% FBS and 1% Penicillin/Streptomycin. Cells were passaged every 2 to 3 days to maintain cells in the undifferentiated myoblast state. On the day of injection, cells were trypsinized, rinsed twice in serum and antibiotic‐free media to ensure no serum or antibiotics were present, and resuspended in media at a concentration of 1 million cells/7 μL. Cells were then stored on ice until injection.

### Animal Care and Surgeries

All procedures were approved by the Institutional Animal Care and Use Committee of the University of Washington. All mice were male C3H (Jackson Labs), ≈3 months old at the time of sacrifice. Since C2C12 cells originated from C3H mice,^19–20^ the injected cells are syngeneic to the host and no immunosuppression was necessary to enhance cell survival. Myocardial infarctions and cell engraftments were performed surgically using a previously established method.^21^ Briefly, each animal was anesthetized with Avertin, and its heart was exposed by thoracotomy using aseptic technique. The left coronary artery (LCA) was permanently ligated, and C2C12 cells were injected into the site of infarction (1 million cells in a single, 7 μL injection). The chest was then closed and the animal was allowed to recover.

Four weeks after infarction and cell injection, mice were sacrificed by ketamine/xylazine overdose followed by intravenous infusion of a saturated potassium chloride solution to arrest the heart in diastole. Hearts were retrogradely perfused with a vasodilator buffer (4 mg/L papaverine and 1 g/L adenosine in PBS), followed by 4% paraformaldehyde, and, if undergoing μCT scanning, the radiopaque dye Microfil (Flowtech) as in Weyers et al^22^ Following perfusion, hearts were excised, fixed in fresh 4% PFA overnight at 4°C, and then stored in 70% ethanol at 4°C.

### Microcomputed tomography Scanning and Image Analysis

Microfilled hearts were imaged in a Skyscan 1076 μCT scanner at 9 μm spatial resolution using the following settings: 40 kV, 70 mA, no filter, 3000 ms exposure, rotation step of 0.5°, 180° scan, and 3 frame averaging. Raw scan data were reconstructed to a 3‐dimensional (3D) slice dataset with an isotropic resolution of 9 μm using the software NRecon V1.6.1.0 (Skyscan), and analyzed using CTan (Skyscan) and Analyze 10.0 (Mayo Clinic) as follows. Samples were thresholded to a level where vessels separated into distinct entities to allow visualization of individual networks, which also limited the analysis to vessels >25 μm in diameter. Nonvascular Microfil (eg, in the atria, aorta, coronary sinus, etc) was digitally removed in Analyze using the “Image Segmentation” module. Then, delineation of graft and/or scar tissue was drawn in CTan: the graft region was manually outlined on several 2‐dimensional (2D) slices via side‐by‐side comparison to histological sections that highlighted the graft and scar (Picrosirius red, and a cardiac troponin T—adult skeletal muscle myosin double stain). The region of interest (ROI) interpolation function in CTan extended the ROI from the manually outlined slices across all slices and produced a 3D representation of the graft or scar. The resulting graft/scar volume was then used to distinguish vessel location (ie, graft/scar or uninjured cardiac tissue) in subsequent analyses. Vessel statistics were obtained using CTan's 2D and 3D statistical calculators: mean number of vessels and average size of vessels were compiled from 2D cross‐sections of each entire volume of interest (VOI) and averaged. Vessel sizes were calculated by converting the cross‐sectional area of each individual object (vessel) to the equivalent circular diameter. Percent vascular lumen (the percent of the total volume of each VOI that is filled with Microfil) was measured within the entire 3D VOI. Individual vessel segmentation to determine vessel identity and branching pattern was performed using Analyze with the same graft/scar VOI imported from CTan. Arterial/venous identity in 3D renderings was assigned by determining the origin of each vascular network (eg, aorta, coronary sinus, etc), as well as by comparing to the previously established anatomy of mouse and rat coronary networks.^23–25^

### Histology

After μCT scanning, hearts were sliced into 4 portions along the transverse axis, processed, paraffin embedded, and cut into 5 μm histological sections, using standard techniques. Briefly, slides were deparafinized in xylenes (Fisher), rehydrated through an ethanol series, washed in PBS 3 times, and incubated in cold methanol with 3% hydrogen peroxide to quench endogenous peroxidase activity. Next, slides were blocked for a minimum of 1 hour with 1.5% Normal Goat Serum (NGS) in PBS (10% NGS for the CD31 antibody). Slides were then incubated with primary antibodies overnight at 4°. The next day, samples were washed 3 times in PBS and incubated with the secondary antibody for 1 hour. Antibody labeling was detected using the peroxidase ABC kit (Vector Labs) followed by either DAB (Vector Labs) or ImmPACT NovaRED (Vector Labs), with the exception of the MY32 antibody, which was detected with either BCIP/NBT (Vector Labs) followed by a brief acid acetone wash (to increase blueness), or VectorRED (Vector Labs). Slides were then dehydrated and coverslipped in permount (Fisher).

Antibodies and lectins were as follows: mouse anti‐adult skeletal muscle myosin heavy chain II (clone MY32, alkaline phosphatase labeled, Sigma A4335, 1:400), rabbit anti‐mouse CD31 (Abcam ab28364, 1:100), rabbit anti‐eNOS (Abcam ab15280, undiluted), *Griffonai* (*Bandeiraea*) *Simplicifolia* lectin I isolectin B4 (Vector labs B‐1205, biotinylated, 1:200), rabbit anticardiac troponin I (Abcam ab47003, 1:50), rabbit anti‐human α‐smooth muscle actin (Abcam ab32575, 1:100), and goat anti‐rabbit::biotin (secondary antibody, Jackson Immunoresearch 111‐065‐003, 1:500).

For picrosirius red staining, samples were deparaffinized in xylenes, rehydrated through an ethanol series, and incubated in a solution of 0.1% sirius red (available from Sigma as Direct Red 80) and 0.1% fast green (Sigma) in 1.3% picric acid (Sigma) before being redehydrated and coverslipped in permount (Fisher).

### Morphometric Analysis

Quantification of vessel lumen sizes was performed using slides immunolabeled for either CD31 or SMA. For each heart, a minimum of 3 microscope fields (40× for CD31, 20× for SMA) were randomly obtained from each of the uninjured, graft, and scar regions. Within each field, vessel lumens were manually identified in Photoshop and painted to highlight the lumenal area. Lumens were counted and their area individually measured using ImageJ.

### Statistical Methods

**Figure 1.**
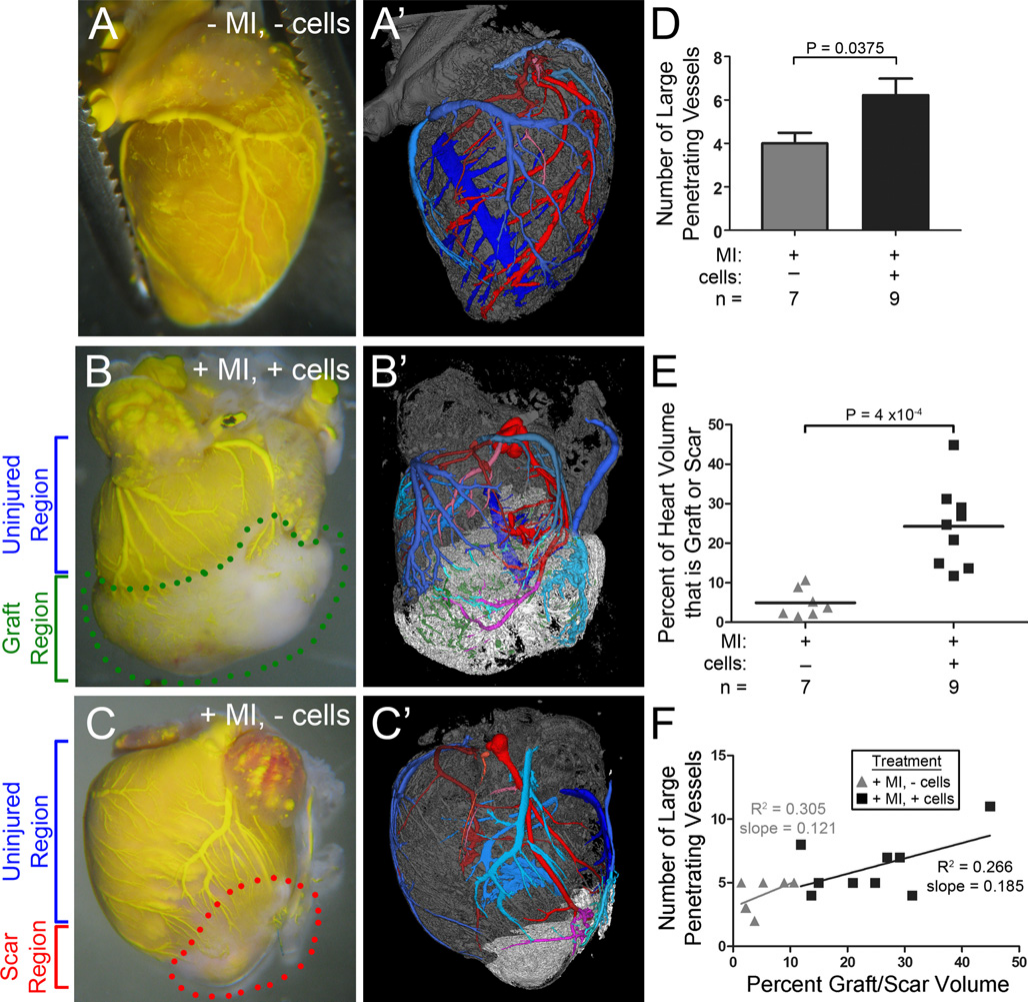
Visualization of the coronary networks reveals vessels within the graft and scar. Brightfield images (A, B, C) and 3D renderings (A', B', C') of hearts perfused with yellow Microfil 28 days after no treatment (A), infarction and cell engraftment (B), or infarction only (C). Graft (B, within green dotted circle) and scar (C, within red dotted line) appear whiter relative to the uninjured myocardium. For 3D renderings (A', B', C'), graft tissue is white, uninjured cardiac tissue is gray. Arteries are shades of red, veins are shades of blue, undefined vessels are green. Vessels change color when entering the graft or scar. Vessels extend from the host tissue into both the graft (B, B') and scar (C, C'). Numerous vessels of various sizes and of both arterial and venous origin are present in both the graft and scar regions. D, More vessels penetrate the grafts than scars. Values are the average total number of large arteries and veins entering/exiting the graft or scar (see Table 1). Error bars are SEM;* P*=0.0375 by unpaired *t* test. E, Graph of graft and scar size, as measured by μCT, shown as percent of the whole 3D heart volume. *P*=4 × 10^−4^ by unpaired *t* test. F, Plot of graft or scar size versus the number of large vessels that invade the graft or scar. Linear regression analysis reveals that larger grafts and scars have a higher number of large invading vessels (including both arteries and veins). The best‐fit lines from both data sets have similar slopes, suggesting that the number of large vessels induced to invade and perfuse a graft or scar area is dependent upon the volume of that area. MI indicates myocardial infarction; μCT, microcomputed tomography

All statistical calculations were performed in Graph Pad Prism (v 5.03). Statistical error is represented as standard error of the mean (SEM). Our data generally follow a Gaussian distribution, passing a D'Agostino‐Pearson normality test in all but a few cases. As such, statistical significance was determined by parametric methods, either by unpaired *t* test (when only 2 groups were compared, as in Figure 1D and 1E), or analysis of variance (ANOVA) followed by a Tukey‐Kramer posttest to determine which specific datasets within the group vary from each other (*P≤0.05, **P≤0.01, ***P≤0.001). Best fit lines to determine potential correlative relationships (Figures 1F and 4D through 4F) were determined by linear regression analysis on each independent dataset.

## Results

### Vascularization of Cardiac Myoblast Grafts

The coronary vasculature was perfused with Microfil at 28 days post–MI and cell engraftment. Gross observation of engrafted hearts (+MI, +cells) revealed branched vascular structures extending from the pre‐existing coronary vascular network into the graft tissue (Figure 1B). Examination of these hearts by μCT analysis revealed that skeletal myoblast grafts developed an extensive vascular network. This network extended through the entire graft, and consisted of both large (>70 μm) and small (≈25 μm; the lower limit of vessels visible by μCT) vessels (Figure 1B'). Tracing the larger branches back to their origin reveals that they do indeed extend from the preexisting coronary network, and both arteries and veins are present in the graft. On average, 2.4±0.3 large arteries extended into each graft, while 3.8±0.9 large veins exited the graft (Table 1, Figure 1D). It is important to note that engrafted tissue is perfused by multiple vessels, rather than being supplied by a single large afferent and a single large efferent vessel. Therefore, the development of graft vasculature is initiated at multiple locations from within the surrounding coronary vascular network.

**Table 1.** More Large Arteries and Veins Penetrate the Graft Than the Scar Region

	Number of Arteries Entering Graft/Scar	Number of Veins Exiting Graft/Scar	n
+MI, −cells	1.86±0.26	2.14±0.46	7
+MI, +cells	2.44±0.29	3.78±0.91	9

Values are mean±SEM. MI indicates myocardial infarction.

The vasculature observed in the scar (+MI, −cells; Figure 1C) was different from the vasculature observed in the grafts (+MI, +cells; Figure 1B). Vascular structures in scars extended throughout the entire scar, consisted of both large and small vessels, and were of both arterial and venous origin (Figure 1C'). However, fewer vessels extended into the scar regions of infarcted hearts than into the graft regions of engrafted hearts (Table 1, Figure 1D). Notably, larger graft or scar volumes (Figure 1E) resulted in more vascular connections to the surrounding tissue (Figure 1F). Thus, the volume of ischemic tissue correlates with the number of vessels required to perfuse the tissue.

### Existing Coronary Arteries Undergo Arteriogenic Remodeling in Response to Cell Engraftment

In the hindlimb ischemia and other vascular remodeling systems, increased flow within a vessel results in the arteriogenic remodeling of the vessel.^26–31^ Similarly, since myoblasts injected into the infarcted myocardium develop into viable tissue^15–16,32^ with a coronary‐derived vascular supply (Figure 1B), we hypothesized that the presence of the graft would induce arteriogenic remodeling within the upstream coronary vascular network. Though our methods cannot distinguish between the de novo formation of new branches or the enlargement of preexisting branches, arteriogenic remodeling of the coronaries would be evidenced by an increase in the number of branches of the left coronary artery (LCA) that are visible by μCT.

**Figure 2.**
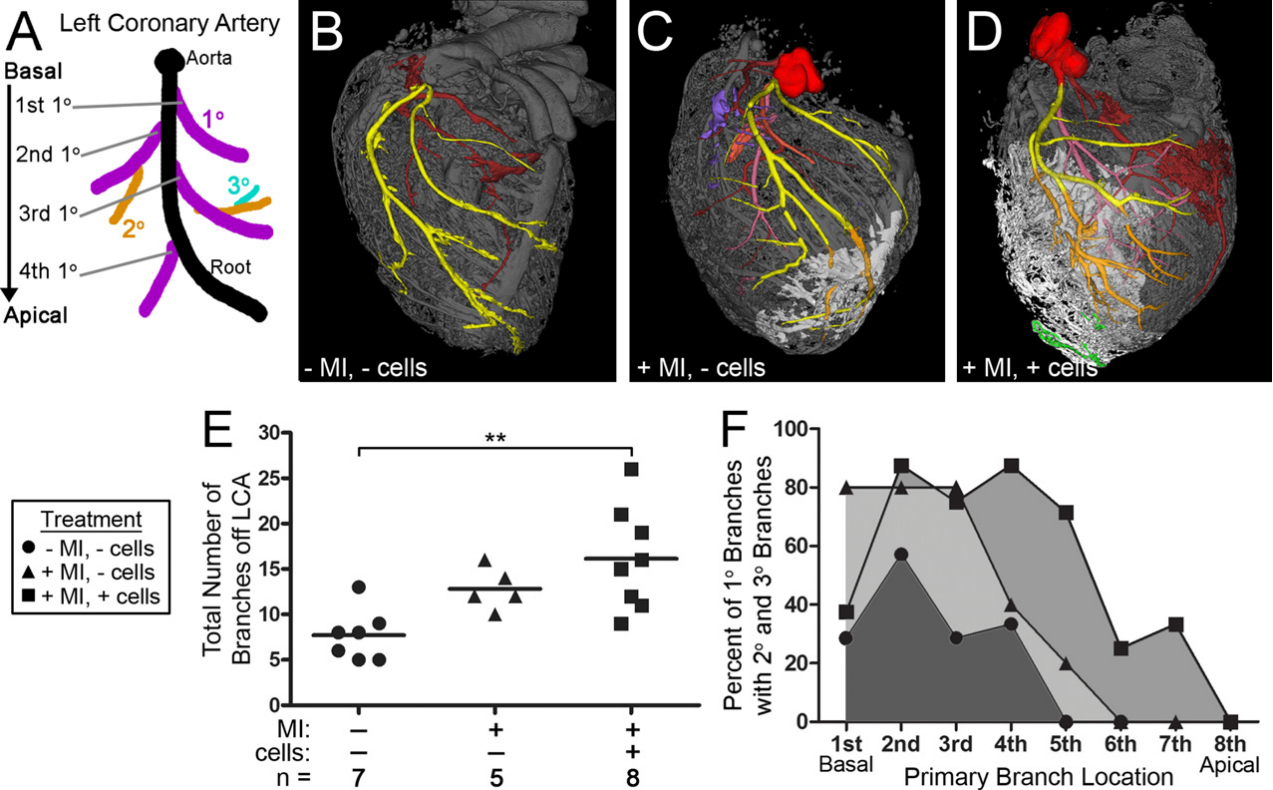
The LCA has an increased number of vascular branches. A, Schematic representation of the LCA branching pattern. The root vessel (defined as the longest continuous vascular branch) is in black. Primary branches (those vessels that directly branch from the root vessel) are in purple. The “first primary branch” is the branch closest to the LCA origin at the aorta, with subsequent branches labeled in order moving down toward the apex of the heart. Secondary branches (arising from the primary vessels) are orange, and tertiary branches (emanating from the secondary branches) are in teal. For simplicity, fewer secondary and tertiary branches are shown in this model than were observed in our experiments. B through D, Representative 3D renderings of heart/graft/scar vasculature. Graft and scar regions are white, uninjured cardiac tissue is gray. Vessels change color when entering the graft. Arteries are shades of red, veins are shades of blue, and undefined vessels are green, with the exception of the LCA, which is highlighted in yellow (in cardiac tissue) or orange (in the graft or scar). LCAs within infarcted (C) and engrafted (D) hearts have more complex branching structures than in control hearts (B). E and F, Quantification of LCA branching. (Note: A smaller number of hearts were analyzed here than in other analyses [Figures 1 through 3] because breaks in the Microfil precluded accurate branching analysis in some hearts.) E, Total number of branches (including primary, secondary, and tertiary) within the LCAs of individual hearts. Line demarcates mean. Engrafted hearts have significantly more branches than control hearts. F, Percent of primary branches that have secondary branches at differing locations along the LCA. Moving down the LCA from basal toward apical, a higher percentage of primary branches at each location have more secondary branches within both the infarcted and engrafted hearts than within the control group. LCA indicates left coronary artery; MI, myocardial infarction.

While the LCA branching pattern is variable from mouse to mouse,^33^ the LCAs in both infarcted and engrafted hearts (+MI, ±cells) appeared to have more complex branching patterns than control hearts (−MI, −cells; Figure 2A through 2D). Quantification of the total number of branches off the root vessel (the longest continuous length of the LCA, Figure 2A) reveals an overall increase in branching in engrafted hearts over control hearts (Figure 2E). In control hearts, the LCA had 4.6±0.4 primary branches (vessels that originate from the root vessel). We observed increased numbers of primary branches originating from the LCAs of both engrafted and infarcted hearts; 5.9±0.5, and 7.0±0.0 (all 5 animals had exactly 7 primary branches), respectively (Table 2, and summarized in total branch count in Figure 2E). The number of secondary and tertiary branches (arising from primary or secondary branches, respectively) was also increased in both infarcted hearts (5.8±1.0) and engrafted hearts (10.3±1.7 branches) as compared to controls (3.1±1.4, Table 2 and summarized in total branch count in Figure 2E). Interestingly, primary branches in infarcted hearts were more likely to have secondary and tertiary branches than in control hearts, regardless of their location along the LCA (Figure 2F). This effect was even greater in engrafted hearts, with primary branches at all but the 2 basal‐most locations being twice as likely as control hearts to have secondary and tertiary branches (Figure 2F). Though the increase in secondary and tertiary branches was greatest toward the apex of engrafted hearts, an increase in branching was also observed at more basal locations, indicating that arteriogenic remodeling occurs throughout the entire coronary network, not just within the areas closest to the graft or scar.

**Table 2.** Number of Branches Off the LCA and LCV

	Arterial/Venous	n	1°	2° and 3°	Total
−MI, −cells	LCA	7	4.6±0.4	3.1±1.4	7.7±1.1
+MI, −cells	LCA	5	7.0±0.0	5.8±1.0	12.8±1.0
+MI, +cells	LCA	8	5.9±0.5	10.3±1.7	16.1±2.0
−MI, −cells	LCV	7	19.0±0.9	15.6±2.3	34.6±2.4
+MI, −cells	LCV	5	17.4±1.9	21.6±6.4	39.0±7.6
+MI, +cells	LCV	5	18.0±1.1	23.8±4.1	41.8±5.0

Secondary and tertiary branch counts are added together. Values are mean±SEM. LCA indicates left coronary artery; LCV, left coronary vein; MI, myocardial infarction.

**Figure 3.**
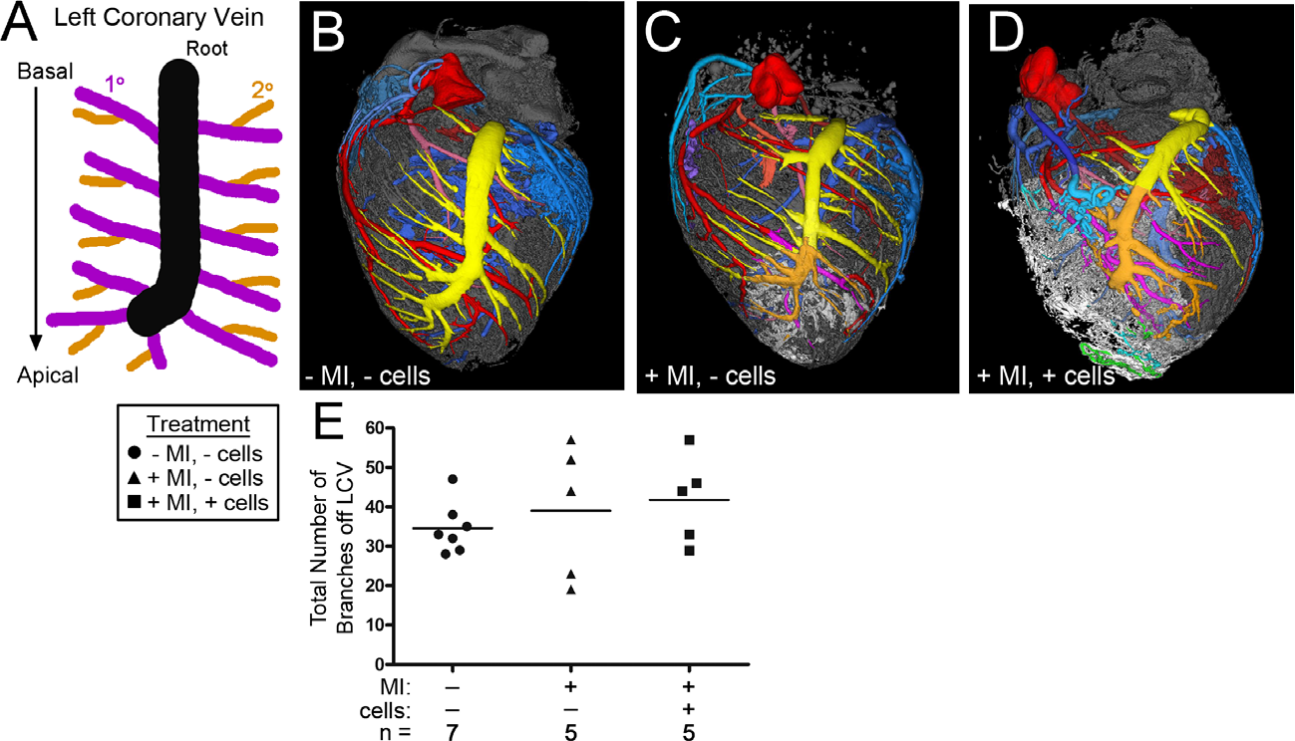
The LCV does not show a significant increase in vascular branching. A, Schematic representation of the LCV branching pattern. The root vessel (black), primary branches (purple), and secondary branches (orange) are defined as in the LCA (see Figure 2 legend). B through D, Representative 3D renderings of heart/graft/scar vasculature. Graft and scar regions are white, uninjured cardiac tissue is gray. Vessels change color when entering the graft. Arteries are shades of red, veins are shades of blue, and undefined vessels are green, with the exception of the LCV, which is highlighted in yellow (in cardiac tissue) or orange (in the graft or scar). LCVs within infarcted (C) and engrafted (D) hearts have more complex branching structures than in control hearts (B). E, Quantification of total number of branches (including primary, secondary, and tertiary) within the LCVs of individual hearts. Line demarcates mean. Despite the visual differences in LCV patterning in engrafted and infarcted hearts as compared to control hearts, there is no significant change in LCV branching frequency. (Note: A smaller number of hearts were analyzed here than in other analyses [Figures 1 through 3] because breaks in the Microfil precluded accurate branching analysis in some hearts.) LCV indicates left coronary vein; MI, myocardial infarction.

Surprisingly, we did not observe a similar change in the number of apparent branches of the venous coronary vessels. Upon gross examination, the left coronary vein (LCV) within infarcted or engrafted hearts did exhibit visible alterations in branch complexity (Figure 3B through 3D); however, these changes did not result in an overall increase in branch number (Table 2 and Figure 3E). These data imply that the presence of myocardial cell grafts promotes arteriogenic remodeling, but not significant venous remodeling.

### Engrafted Tissue is Characterized by Fewer and Smaller Vessels Than Uninjured Myocardium

**Figure 4.**
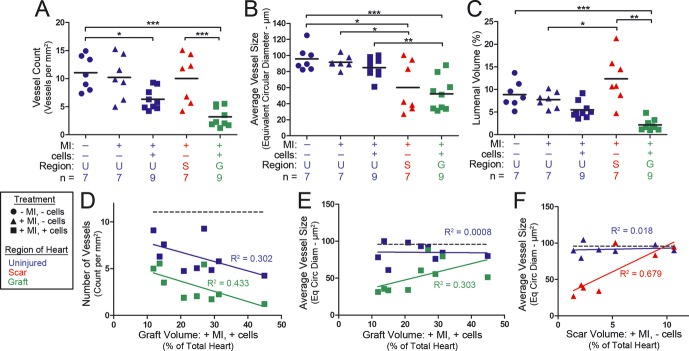
Grafts have smaller and fewer vessels than control hearts. A through C, Lines represent the average of the plotted data. A, Average vascular density (ie, the number of vessels per mm^2^) within individual hearts, as determined by μCT analysis. Engrafted hearts have fewer vessels both within the graft as well as within their uninjured regions. B, Average vessel size (ie, the equivalent circular diameter of the lumen). Graft and scar regions have smaller average vessel sizes relative to uninjured regions. C, Percent lumenal volume (ie, the proportion of tissue occupied by vascular lumen). Grafts have a lower vascular lumenal volume than control hearts. Scar regions have higher lumenal volumes than the corresponding uninjured regions. D through F, Plots of graft volume (D and E) or scar volume (F) vs the vascular density (D) or average vessel size (E and F). Linear regression analysis was performed on individual datasets to determine best‐fit lines and their corresponding R^2^ values. D, Graft volume (as percent of total heart volume) correlates to vascular density such that larger grafts have fewer vessels both in and outside the graft. Dotted line represents the average vascular density within control hearts (see A). E and F, Vessel size within the graft (E) and scar (F) correlates to graft or scar size such that bigger grafts and scars have bigger vessels. Dotted lines represent the average vessel size within control hearts (see B). μCT indicates microcomputed tomography; MI, myocardial infarction; U, uninjured; S, scar; G, graft.

To further characterize the graft vasculature we compared vessels with >25 μm diameters within scars (the scar regions of +MI, −cells hearts, outlined in Figure 1C), grafts (the graft region of +MI, +cells hearts, outlined in Figure 1B), and the uninjured myocardium within all 3 treatment groups (−MI, −cells; +MI, −cells; and +MI, +cells). Scar regions exhibited a similar vascular density to that of uninjured myocardium (Figure 4A). In contrast, graft regions exhibited less than half the vascular density of either scar regions or uninjured hearts (Figure 4A). Interestingly, the uninjured region of engrafted hearts also had a lower vascular density than that of uninjured hearts (Figure 4A), suggesting the presence of engrafted cells induces pruning or a decrease in vessel size which causes the vessels to become smaller than the detectable range of our μCT analysis.

**Figure 5.**
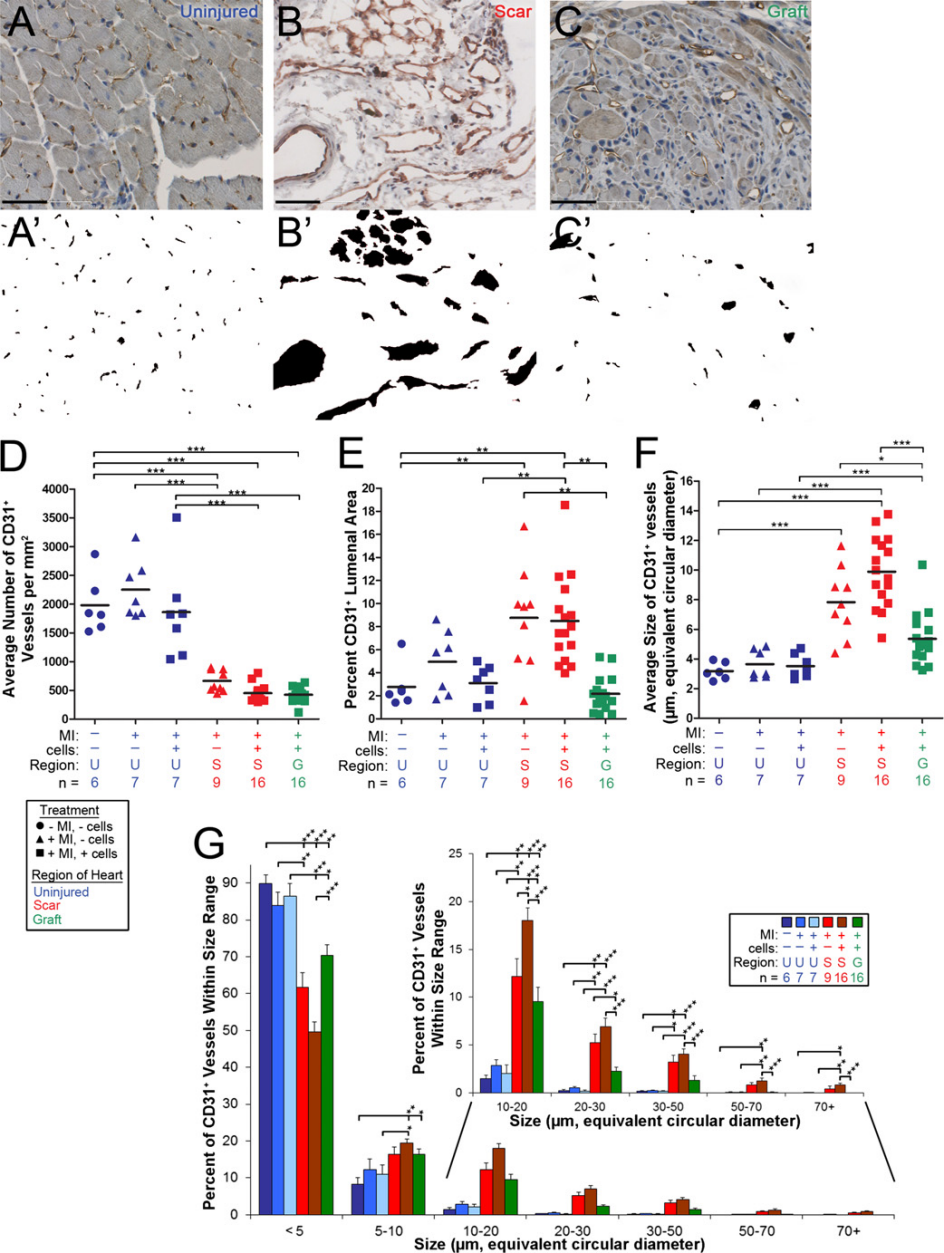
There are fewer and larger CD31^+^ microvessels in the graft. A through C, Immunohistological detection of the endothelial marker CD31 to identify vessels within uninjured tissue (A), the scar (B), or graft (C). Scale bars=50 μm. CD31^+^ lumens are highlighted (A', B', C') to allow for easier visualization of the vascular organization within the tissue. Vessels within the uninjured tissue (A) are mostly capillary‐sized and evenly spread across the tissue. Vessels within the scar (B) and graft (C) are not as evenly spaced throughout the tissue, leaving some areas with no vessels. Graft and scar vessels are also larger in size than in uninjured heart tissue, with those in the scar being extremely large and sinusoidal in appearance. D through G, Morphometric analysis of CD31^+^ lumens. Statistical significance was determined by ANOVA, followed by Tukey posttests to determine which groups differed within each set. D through F, Solid lines are averages. D, Graph of vascular density (average number of CD31^+^ vessels per mm^2^). Graft and scar regions have lower vascular densities than uninjured regions. E, Graph of percent lumenal area. The scar regions have higher percent lumenal areas than the graft or uninjured regions. F, Graph of average CD31^+^ vessel size. Vessels within the graft are, on average, larger than those in uninjured myocardium. In scar regions, this is exaggerated such that vessels are, on average, more than twice the size of those in uninjured myocardium. G, Histogram of the distribution of CD31^+^ vessel sizes. Error bars are SEM. Scar and graft regions have lower percentages of small vessels, and higher percentages of larger vessels than uninjured myocardium. ANOVA indicates analysis of variance; MI, myocardial infarction.

The average size of vessels was smaller in both the graft and scar regions than within uninjured myocardium (Figure 4B). Additionally, the percent lumenal volume was lower in the graft than in the uninjured myocardium (Figure 4C). On the other hand, scar regions, despite having similar vascular density (Figure 4A) and smaller vessel size (Figure 4B), have greater percent lumenal volume than uninjured myocardium (Figure 4C). This may be a result of the extremely large “sinusoidal‐like” vessels observed within the scar (see Figure 5B). Overall, these data demonstrate that the cell grafts are vascularized as expected, but the vascular organization observed in grafts is significantly different than that in uninjured myocardium.

To determine whether graft or scar volume correlates with the organization of graft vasculature, we compared the size of the graft and scar (Figure 1E) to the vascular parameters described above. The vascular density in the graft decreased with increasing graft volume (Figure 4D). Interestingly, this trend was also observed in the uninjured myocardium of the same hearts, again suggesting that the presence of graft cells induces the surrounding myocardium to undergo pruning or downsizing (Figure 4D). In the absence of cell engraftments, however, there was no correlation between vessel density and scar volume (data not shown). Vessel size, on the other hand, was found to positively correlate with both graft and scar volume: larger grafts and larger scars contained larger vessels (Figure 4E and 4F), but this effect was not observed in the surrounding uninjured myocardium (Figure 4E and 4F).

### Myocardial Grafts Are Vascularized With Both Macro‐ and Microvessels

Vessel analysis by μCT is limited to vessels larger than 25 μm in diameter. Therefore, in order to characterize vessels of all sizes we performed immunohistological analysis. Vascular lumens were identified by immunoreactivity to the endothelial marker CD31 (Figure 5A through 5C; immunohistochemistry for eNOS and isolectin B4 gave similar results [data not shown]). While CD31^+^ vessels in the uninjured myocardium are small (<7 μm lumenal equivalent circular diameter) and of uniform size and distribution (Figure 5A), vessels in the graft have variable sizes (≈1 to 70 μm) and are unevenly distributed throughout the tissue (Figure 5C). In the scar region, these effects are more exaggerated: some vessels are extremely large (>70 μm) and “sinusoidal,” and vessels are unevenly distributed such that there are large gaps in the vascularization of the tissue (Figure 5B). Despite the differing vascular organization, this analysis does confirm that capillaries are present within both the graft and scar post‐MI.

Quantification of the histological results reveals that there is a lower vascular density in the graft and scar regions as compared to the uninjured myocardium (Figure 5D). Despite this decrease in vessel number, vascular lumens occupied a much greater fraction of tissue area in the scar (8.8%) than in noninfarcted tissue (2.8%), due to the presence of the sinusoidal vessels (Figure 5B and 5E). Within engrafted tissue, however, there were fewer sinusoidal vessels, resulting in a luminal area similar to uninjured myocardium (Figure 5C and 5E). Additionally, vessels in both the graft and scar are larger than in uninjured myocardium (Figure 5F and 5G). Vessel size in uninjured cardiac tissue is distributed such that almost 90% of vessels are in the smallest size range (0 to 5 μm in diameter), and less than 1% of all vessels have diameters over 20 μm (Figure 5G). In the graft, however, this distribution is shifted such that only 70.4% of vessels are in the smallest size range, and 3.6% are over 20 μm (Figure 5G). Vessel sizes within the scar are shifted even more; the smallest vessels represent less than 62% of the population, and vessels of more than a 20 μm diameter account for more than 9.5% of all vessels (Figure 5G). Thus, the microvasculature of the engrafted tissue remodels into a network of both large and small vessels, with a vascular pattern that more closely resembles that of the uninjured myocardium than the scar region.

### The Graft Vasculature Contains Smooth Muscle‐Encoated Vessels

**Figure 6.**
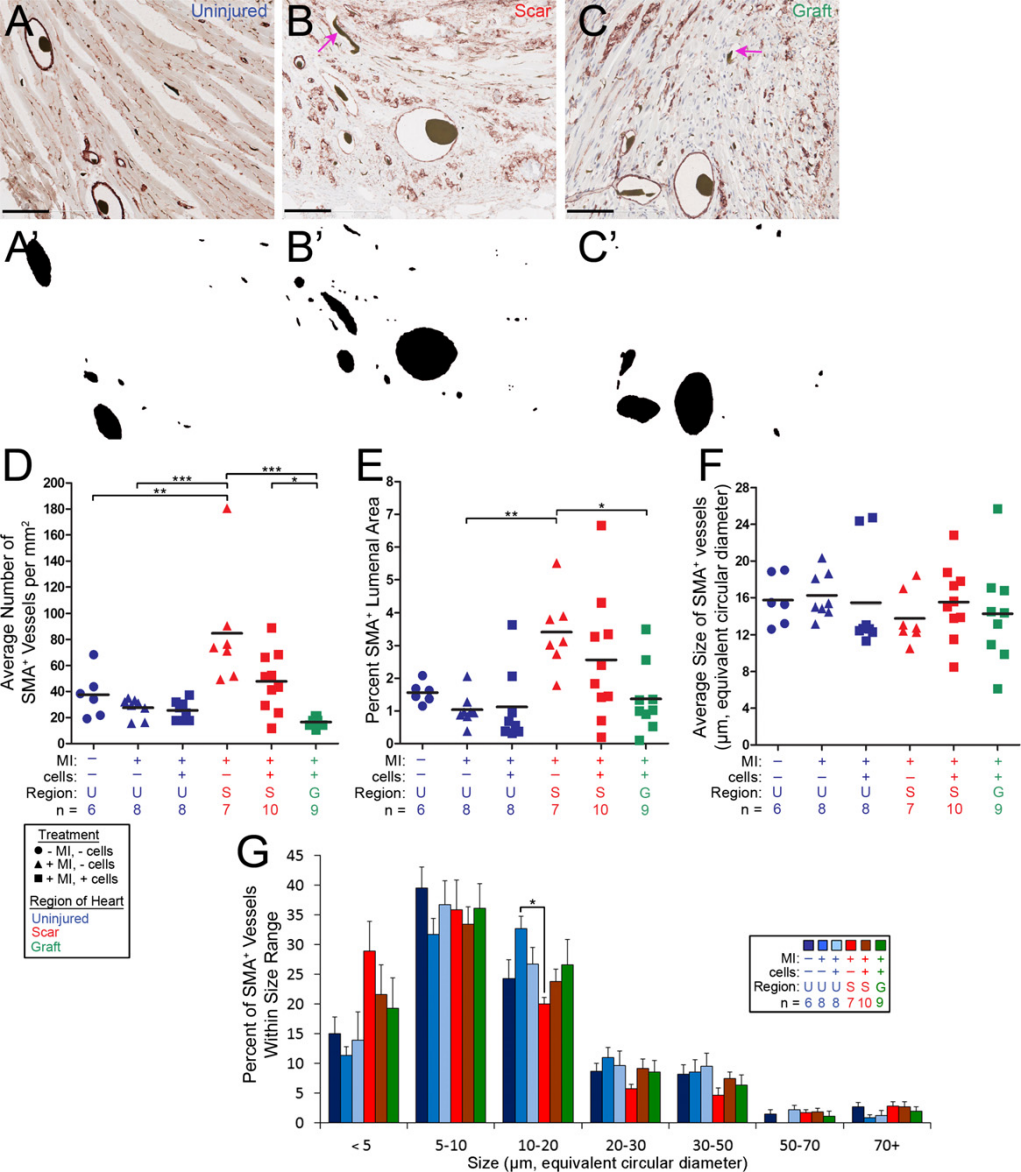
Histology reveals SMC coated vessels in the graft and scar. A through C, Immunohistochemical labeling of SMCs with the marker SMA to identify vessels with SMC coats in the uninjured tissue (A), scar (B), and graft (C). Scale bars=100 μm. SMA^+^ lumens are highlighted (A', B', C') to allow for easier visualization of the vascular organization within the tissue. SMC coated vessels are present within all 3 regions. Notice the large vessel‐like structures in the graft and scar that were perfused with Microfil (black clump) but are lacking an SMC coat (pink arrows). D through G, Morphometric analysis of SMA^+^ lumens. Statistical significance was determined by ANOVA, followed by a Tukey posttest to determine which groups differed within each set. D through F, Solid lines are averages. D, Graph of smooth muscle coated vessel density (average number of SMA^+^ vessels per mm^2^). The graft has almost half the number of SMC coated vessels compared to control hearts, while scar regions in nonengrafted hearts have almost twice the number of SMC coated vessels as control hearts. Interestingly, scars in hearts without cell engraftments have a higher number of SMA^+^ vessels as those in hearts with cell engraftments. Although this statistic did not reach significance, it suggests that the presence of the graft reduces the number of SMC coated vessels within the neighboring scar region. E, Graph of the percent SMA^+^ lumenal area. A higher percent lumenal area is occupied by SMA^+^ vessels in scar regions than within the graft or uninjured regions. F, Graph of average SMA^+^ vessel size. The average size of SMA+ vessels does not change significantly between uninjured regions and the graft or scar. G, Histogram of the distribution of SMA^+^ vessel sizes. SMC coated vessels within uninjured, scar, and graft regions exhibit a similar size distribution. SMC indicates smooth muscle cell; SMA, smooth muscle α‐actin; ANOVA, analysis of variance; MI, myocardial infarction.

In order to form a hierarchical network, some vessels within the graft must remodel into arterioles or venules, and must therefore develop smooth muscle coats. Immunodetection of smooth muscle α‐actin (SMA) revealed that smooth muscle cell (SMC)‐coated vessels are present within both the graft and scar region (Figure 6B and 6C). The large sinusoidal vessels observed in the scar (see Figure 5B) typically lacked SMC coats (Figure 6B). Quantification of SMA immunohistochemistry reveals that there was approximately half the density of SMC‐coated lumens within the graft as compared to the uninjured myocardium (Figure 6D). The scar, on the other hand, had more than twice the number of SMC‐coated vessels (Figure 6D) and they comprised a higher percentage of tissue area (Figure 6E) than uninjured regions. Despite the differences in SMA^+^ vessel density and lumenal area, the average vessel sizes and size distributions of SMC‐coated vessels within the graft, scar, and uninjured regions were similar (Figure 6F and 6G).

## Discussion

This study demonstrates that the coronary network is capable of remodeling and developing a complex vascular network to support tissue engrafted into the infarcted murine myocardium. Twenty‐eight days post‐MI and implantation of skeletal myoblasts, vessels extend from the preexisting coronary tree to perfuse the engrafted tissue. Furthermore, the graft vasculature is composed of vessels of various sizes, some of which have recruited SMC coats. This implies that vascular remodeling occurs within the graft at both the conducting and microvascular levels. Strikingly, the presence of the cell graft also induces arteriogenic remodeling of the coronary vasculature distal to the graft, resulting in an increase in the number of arteries visible by μCT.

Given the limitations in visualizing the mouse coronaries throughout the experimental time‐course, we cannot rule out the formation of new branches in our myocardial engraftment model. Evidence from the hindlimb ischemia,^27,34–37^ carotid ligation,^30–31^ and chronic vasodilation^28–29^ models implicates flow‐induced remodeling, driven by an increase in fluid shear stress, in the enlargement of vessels. In these systems, however, no new vessels are formed. Instead, preexisting vessels are remodeled to accommodate changes in blood flow.^26,38^ In the coronary vasculature, numerous small vessels are known to branch from the LCA (mouse) and left anterior descending artery (human) which would not be visible at the resolution of μCT,^33,39^ but could enlarge to become the “new” vessels observed by μCT post‐MI. In light of our data, we suggest that it is more likely that the newly apparent vessels arise via remodeling of these preexisting branches than through the de novo development of vessels. We refer to this change in arterial structure as “arteriogenic remodeling.” Interestingly, since these preexisting small vessels within the coronaries have been described in both mouse^33^ and man,^39^ our results raise the intriguing possibility that, in response to a stem cell graft, the human heart may also be able to undergo arteriogenic remodeling.

While increased flow acts as the stimulus for arteriogenic remodeling in other experimental vascular remodeling systems,^28–31,28–37^ in our engraftment model, we observed an increase in apparent arterial branching along the entire length of the LCA distal to the graft (Figure 2). Some of these newly apparent arteries did not appear to directly perfuse the graft. Thus, models of flow‐induced remodeling cannot account for all the observed vascular changes, indicating that diffusible factors are also likely to mediate arteriogenic remodeling in the myocardium. Studies in the hindlimb have implicated multiple growth factors, including fibroblast growth factor, platelet‐derived growth factor, placental growth factor, and vascular endothelial growth factor (VEGF) as critical mediators of this process.^34,40–41^ Consistent with this, the experimental antagonism of the angiogenic factors hedgehog^42^ and VEGF^43^ has recently been attributed to pruning of cardiac vessels. We observed that the vascular density within uninjured myocardium was decreased by almost 50% in the presence of a graft (Figure 4A), an effect that correlated with graft size (Figure 4D). Thus, the presence or absence of diffusible angiogenic and arteriogenic factors may affect tissue adjacent to the graft.

Our analysis also provides insight into the formation of a vasculature within the graft. To establish functional blood flow within the graft, vessels must organize into a hierarchically ordered vascular circuit. Canonical models implicate angiogenesis in the initial establishment of a vascular network, followed by the enlargement and muscularization of select capillaries through SMCs encoatment.^12,14,44–45^ At 28 days postinfarction and cell injection, capillaries and arterioles (small muscular arteries) were present within the graft (Figures 5 and 6). Since the graft is comprised of newly implanted cells devoid of a preformed vasculature, the observed vasculature must be newly formed. While we cannot determine the extent of maturation into a hierarchical network without higher resolution 3D vascular imaging, the presence of variably sized vessels in this newly implanted tissue is consistent with the idea that complex vessel growth and remodeling mechanisms are occurring within the graft. Unfortunately, since our data only encompass a single time‐point post‐MI, we cannot confirm whether these vascular growth mechanisms are truly occurring. However, grafts were supplied by multiple afferent arterial branches from the LCA (Figures 1 and 2, Table 1), indicating that vascular growth into the graft is initiated at multiple locations. Interestingly, histological evidence demonstrated that grafts exhibited a lower percentage of small vessels as compared to untreated myocardium (Figure 5G). This discrepancy in size distribution may indicate aberrant or poorly regulated remodeling such that a disproportionate number of capillaries are enlarged in the graft. Alternatively, the growth and remodeling of graft vasculature into an efficient hierarchical network may not be complete by 28 days post‐MI and cell injection, and if this study is extended to a later time‐point, the observed size discrepancies may be resolved. The basis for remodeling these small vessels, other than the requirement to recruit smooth muscle cells,^44^ is an opportunity for exploration at a molecular level.

This work has also raised additional questions regarding the establishment of vasculature within engrafted tissue. There are several observations resulting from our data that do not have clear explanations. For example, many of the mechanistic causes of the relationships between graft size, vascular density, and vascular size are unclear (ie, larger grafts exhibit fewer vessels both inside and outside the graft, but larger grafts also have larger vessels, Figure 4D and 4E). Therefore, there is still much to be understood about how the vasculature is established and the mechanisms by which the new and existing vasculature is organized. These and other questions remain to be answered in future work.

While little is known about venous remodeling or the mechanisms regulating coordinated remodeling between arteries and veins, it is interesting to note that our data demonstrated that remodeling of coronary veins did not occur in parallel to arterial remodeling (Figure 3). This is surprising, as we had expected coordinated remodeling would be required to establish a balanced afferent and efferent system. This may indicate that venous remodeling occurs by a different mechanism, or alternatively, that veins are more adaptable to changes in blood flow as compared to arteries.

In summary, this study demonstrates that the coronary vasculature undergoes arteriogenic remodeling to support the vascularization, perfusion, and survival of myocardially‐implanted cell grafts after infarction. Understanding the molecular mechanisms mediating both the arteriogenic and angiogenic remodeling processes will be important to the successful efforts to create functional myocardial cell grafts of other more therapeutically relevant cell types (eg, iPS‐derived cardiomyocytes). Importantly, we propose that the induction of a similar arteriogenic remodeling process in humans could potentially allow for the growth of vessels around coronary occlusions preinfarction, as well as provide an alternative to surgical procedures to revascularize ischemic hearts following infarction.
